# Monetary policy, financial shocks and economic activity

**DOI:** 10.1007/s11156-022-01045-z

**Published:** 2022-02-20

**Authors:** Anastasios Evgenidis, Anastasios G. Malliaris

**Affiliations:** 1 Newcastle University Business School, Newcastle University, 5 Barrack Rd, Newcastle upon Tyne, NE1 4SE UK; 2 Quinlan School of Business, Loyola University Chicago, 16E Pearson St, Chicago, IL 60611 USA

**Keywords:** Financial shocks, Credit, Spreads, VAR, Financial crisis, Monetary policy, E50, E52, E58

## Abstract

This paper contributes to a fuller understanding of macroeconomic outcomes to financial market disturbances and the central bank’s role in financial stability. Our two major contributions are conceptual and econometric. Conceptually, we introduce phases of the business cycle and econometrically we employ Bayesian VARs. We document that a shock that increases credit to non-financial sector leads to a persistent decline in economic activity. In addition, we examine whether the behavior of financial variables is useful in signaling the 2007–2009 recession. The answer is positive as our BVAR generates early warning signals pointing to a sustained slowdown in growth. We propose that the expansion phase of the business cycle can be subdivided into an early and a late expansion. Based on this distinction, we show that if the Fed had raised the policy rate when the economy moved from the early to late expansion, it could have mitigated the severity of the 2007–2009 recession.

## Introduction

Suppose that four years before the Lehman Brothers bankruptcy and the eruption of the Global Financial Crisis, the Federal Open Market Committee (FOMC) had the perfect foresight to recognize that a housing bubble was emerging. It is reasonable to argue that monetary policy would still evolve as it did with small and repeated increases to the Federal funds rate (FFR) that stood around 1% in June 2004 and reached about 5.5% in September 2006. One reason for monetary policy to have ignored the possibility of a housing bubble was the mindset of central bankers labeled as the Jackson Hole consensus that disregarded asset bubbles and favored cleaning instead of leaning against such asset bubbles.

Rajan (2005) reflected that the financial system can diversify small shocks but remains exposed to larger systemic shocks that may result from large declines in asset prices and aggregate liquidity. Furthermore, the author encouraged economists to understand the linkages between credit conditions and the real economy given that financial markets have grown substantially and their stability has become more fragile. Rajan’s trepidations about increasing risk-taking were effectively disregarded by most economists and policy makers whose macroeconomic methodologies underestimated the impact of financial shocks on the real economy.

The Global Financial Crisis of 2007–2008 and its U.S. Great Recession compelled economists to re-examine the impact of financial variables on output and employment since the bursting of the housing bubble is widely viewed as the archetypal financial shock that triggered the crisis. The purpose of this paper is to present detailed empirical evidence that contributes to a greater understanding of macroeconomic outcomes to financial market disturbances towards the following aspects.

First, we investigate the responses of a large set of variables that includes standard macroeconomic aggregates, credit and financial variables, to financial market disturbances originated mainly in the domestic banking sector. In this context, we also seek to explore whether credit expansions may be followed by substantial declines in economic growth and if so, whether the decline in economic activity is due to the systematic monetary policy contraction that the credit shock elicits. Furthermore, our study adds to the related literature by uncovering the importance of various credit and financial shocks in explaining economic activity fluctuations. To answer these questions, we embed rich information from various dimensions of credit and financial markets into a state-of-the art large scale Bayesian VAR model (BVAR). We call this our full model. The BVAR tools used to examine these issues are impulse responses and historical decompositions.

Most studies on the impact of credit markets on economic activity either focus on credit growth, or incorporate information in credit spreads. Both strands of literature have looked at either single-equation models that do not consider endogeneity issues (Lopez-Salido et al. 2015; Krishnamurthy and Muir 2016) or multi-equation models (Gilchrist and Zakrajsek 2012; Gertler and Karadi 2015 and Mian et al. 2017), which however are small scale VARs, not being able to incorporate more than five variables at a time. Our study is closer to Brunnermeier et al. (2021), however, as opposed to their study, our approach uses a more comprehensive data on credit and financial markets to capture spillovers between the financial sector and the macroeconomy, a simpler identification strategy of just one structural shock, that is a credit supply shock, while we also focus on disentangling the significance of each individual credit and financial shock in driving economic growth throughout the sample.

Next, we narrow our focus and investigate whether credit and financial variables are useful in signalling the recession of 2007–2009 and if so, which variables might have given early signals of a bubble formation or exuberance in the housing market. We use our BVAR full model to conduct out-of-sample forecasts and we compare BVARs with different sizes to examine whether financial variables, including spreads, credit aggregates and asset prices, are practically helpful in providing short-term forecasts of the 2007–2009 recession. Our advantage with respect to Brunnermeier et al. (2021) comes from our use of a richer specification that includes information on stock markets, house prices, financial volatility, consumer sentiment and exchange rates, that can enhance the predictive power of our model.

Second, having demonstrated that our full model is able to offer advance warning signals of the 2008 recession, we use it to address policy relevant issues. We are interested in exploring the trade-off between the traditional mandates of monetary policy, i.e. output and inflation stability, versus addressing risks to financial stability thus reducing the probability of a recession. This challenge of the central bank is explored in detail in Evgenidis and Malliaris (2020) and Bhar and Malliaris (2021). In this paper, we propose that policy makers need to consider business cycle phases when they design monetary policy. We therefore employ the idea of subdividing the expansion phase (from trough to top) into an early expansion and a late expansion. Haberler (1960) devotes an entire book discussing phases of business cycles and economic theories proposed to describe economic developments associated with these phases.

Late expansions often merge with upper turning periods. Within an expansion phase, we hypothesize that as the expansion matures and the economy transitions to the later period of the business cycle, the role of monetary policy becomes more challenging. The reason for this challenge is that once an economy has reached a level close to full employment, risks of potential inflation grow and trade-offs between maximum employment and price stability become more testing. Put differently, during the late expansion, the economy has grown sufficiently to be close to full employment with prices remaining stable, therefore, it is a period when potential risks of inflation emerge, and the financial stability of the economy arises as a central issue. If we consider the actual timing when the Fed reversed its policy from expansionary to mildly contractionary, such timing might be late. The question that we ask is therefore, if the long expansion phase can be subdivided into an early expansion and a late expansion period, could the Fed during the early expansion focus on its traditional mandate and during the late expansion, specifically at the dawn of the late expansion period, switch to addressing risks to financial stability? To empirically examine these reflections, we proceed as follows.

Initially, we propose a novel way to identify the late expansion of the business cycle by adopting a time varying VAR model (TV-VAR) with stochastic volatility. The analysis is close to Cogley and Sargent (2005), Benati (2008), and Akram and Mumtaz (2019) who provide evidence on the Great Moderation in the US, the UK and Norway. However, in contrast to these papers, our investigation adds to the literature by exploring the time-varying trends and volatilities of macroeconomic and credit variables to uncover whether early expansion phases and later periods are distinct phases of the business cycle. We place particular emphasis on the period between the dot-com bubble and the 2007–2008 crisis. Ideally, we would like to see evidence of potential structural shifts that took place in 2003–2004, before the Fed began increasing the policy rate. Based on the separation of periods that is dictated by the TV-VAR model, we construct counterfactual policy experiments to investigate whether a policy of leaning against the built-up of financial imbalances at the dawn of the late expansion period, could have lessened the consequences of the 2007–2009 Great recession.

Our main results are as follows. We find that a shock that increases credit to the non-financial sector leads to a persistent decline in economic activity. Additionally, historical decompositions that allow us to obtain a broader picture of the impact of various financial shocks on the economy through time show that the idiosyncratic shocks of credit and asset price variables contribute substantially to the fluctuations of industrial production (IP) over the entire sample, including both the 2001 and 2008 recessions. Next, our BVAR forecasts reveal the following interesting finding. When we consider a model without any credit variables, we observe that it does a poor job in forecasting the 2008 recession as it never recognizes the crisis while it consistently predicts that economic growth will return to near pre-crisis levels. On the contrary, we find that the addition of credit aggregates, interest rate spreads and asset prices can offer significant advance warning signals of the upcoming recession and enhance recognition of its severity.

Next, we find strong empirical evidence for two distinct periods, before and after 2003, that subdivide the expansion phase of the business cycle into the early expansion phase and the late expansion period. The long-run means show that there is a substantial drop in IP and consumer sentiment as well as a notable rise in inflation rate, the policy rate and the credit to non-financial sector after 2003. Similarly, for the same period, we find evidence of increases in the volatility of IP, inflation and consumer sentiment. These dynamics of the volatilities can be roughly described as a transition from a low to high volatility regime between the period before and after 2003. Counterfactual simulations suggest that leaning against the build-up of financial imbalances helps to moderate the increase in asset prices in the period before the crisis. We find that a policy tightening against rising asset prices applied during the early stages of the late expansion period, could have mitigated the severity of the 2008 recession, without significant costs in terms of high unemployment, low inflation and growth effects of asset prices.

The remainder of the paper is organized as follows. Section two provides a brief literature on the impact of credit and financial conditions on the real economy and next, it turns attention to the business cycle paradigm that motivates our respective hypothesis. Section three discusses the econometric models that we use. Section four presents and discusses the results while section five concludes.

## Related literature

In the first part of our paper, we aim to answer the following research questions. How do core macroeconomic, credit and financial variables respond to financial market disturbances? What is the contribution of various financial and credit shocks in driving economic growth? Also, are credit and financial variables helpful in predicting future output growth? All these questions tie together under a common framework that is related to the impact of credit and financial fluctuations on the real economy.

The Global Financial Crisis of 2007–2008 intensified economic research related to the relationships that arise between credit conditions and their impact on the aggregate real economy, both by revisiting earlier contributions on the role of aggregate credit and by extending these past contributions to incorporate new realities from the financial crisis.

In the monetarist view of Friedman and Schwartz (1963) but also in the more recent Neo-Keynesian synthesis (e.g. Woodford 2003), macroeconomic outcomes are largely independent of the performance of the financial system. On the other side, studies such as Bernanke (1983, 1993), Gertler (1988) or more recently Battacharya et al. (2011), Eggertsson and Krugman (2012) and Brunnermeier et al. (2013), have argued that credit conditions can have a powerful, distinct, and sometimes even dominant effect on the macroeconomy. Increased leverage raises the vulnerability of economies to financial shocks and can lead to more pronounced confidence shocks and expectations swings. Financial accelerators effects are also likely to be stronger when balance sheets are larger. Such effects could be more pronounced in a systemic crisis due to banking failures and asset price declines.

On the one hand, there have been many studies on the credit fluctuations-growth nexus that link credit deepening and the role of financial intermediation, with economic growth (King and Levine 1993; Rancière et al. 2008). Levine (2005) surveys a large literature that uses post-World War II panel data. In a nutshell, these contributions indicate that countries with increased financial market deepening, a higher credit to GDP ratio, or higher stock market capitalization, experience more rapid growth. Empirical evidence for the beneficial effects of financial booms is also provided by Rousseau and Wachtel (2017) who used historical data for the period 1870–1929 and confirmed that financial deepening contributes to positive growth effects. Rousseau and Wachtel (2009) however in another study found that these positive growth effects due to financial deepening have been milder since 1985 which concurs with a rise in the occurrence of financial crises.

On the other hand, significant contributions have supported the view that credit growth is often associated with banking crises with severe effects on the real economy. Jorda et al. (2014) showed that the growth of mortgage credit has important implications for the sources of financial fragility, especially in advanced economies and therefore for the design of macroeconomic policies. The authors also demonstrated that mortgage credit has increasingly left its mark on business cycle dynamics. In the post-WWII period, the aftermath of mortgage booms gone bust is marked by significantly slower growth rates, regardless of whether a financial crisis occurred or not. In a similar spirit, Jordà (2013) using data on 14 advanced countries documented that the aftermath of leveraged booms is associated with slower growth, investment spending and credit growth than usual. Moreover, if the recession coincides with a financial crisis, these effects are compounded and accompanied by pronounced deflationary pressures.

Mian et al. (2017) suggested two broad hypotheses that relate household debt to business cycles. On the one hand, the ‘credit demand hypothesis’ predicts a positive relationship between current household borrowing and future income. In this view, household borrowing is driven by productivity or technology shocks that increase expected future income, leading to higher consumption and borrowing in anticipation. On the other hand, the ‘credit supply hypothesis’ posits that higher household borrowing is driven by an expansion in the availability of credit. In the presence of certain frictions such as nominal rigidities or monetary policy constraints, households may make the economy susceptible to “excessive” credit growth, thus leading to an eventual slowdown in GDP growth. Mian et al. (2017) showed that an increase in the household debt to GDP predicts lower subsequent economic growth and increases unemployment, in a panel of 30 countries. In addition, the authors suggested that there is a global household debt cycle that can predict the intensity of the global recession in the post-2008 period given the large increase in household debt in the mid-2000s. Countries with a household debt cycle that is linked more with the global cycle, experience a sharper fall in economic growth in response to rise in household debt.

Finally, Aikman et al. (2020) show that the effects of financial conditions and monetary policy on U.S. economic performance depend nonlinearly on nonfinancial-sector credit. When credit is below its trend, an impulse to financial conditions leads to improved economic performance and monetary policy transmission works as expected. By contrast, when credit is above trend, a similar impulse leads to an economic expansion in the near term, but then a recession in later quarters.

### Business cycles and monetary policy

In the second part of our paper, we ask whether a monetary policy dependent on the business cycle phases, particularly the subdivision between an early expansion and late expansion periods, could have yielded noticeable output gains in the post 2007–2008 period. It is therefore imperative to offer a discussion on the business cycle paradigm that motivates our empirical analysis.

Officially, the National Bureau of Economic Research (NBER) closely monitors the evolution of the U.S. economy and decides business cycle dating. It also lists expansions and contractions, where contractions mean recessions.^1^ Note that in the NBER methodology, there are no turning periods. When economists talk about the general business cycle with its four phases: expansion, upper turning period, recession and lower turning period, the NBER methodology reduces the upper and lower turning periods to just one month (most often one quarter) prior to the recession and one month (or one quarter) prior to the recovery.

There is a large literature nicely summarized in Haberler (1960) that proposes various hypotheses about turning periods. Expansions do not abruptly become recessions nor do recessions suddenly end to be followed with expansions. Haberler (1960) unwearyingly reviews “real”, “monetary” and “other” theories of business cycles where upper turning periods and lower turning periods are distinct phases of the business cycle. Minsky (1986) revived this literature in his hypothesis that the stability during the expansion period often leads to instability during the upper period that in turn produces the crisis and the recession or depression.

**Fig. 1:**
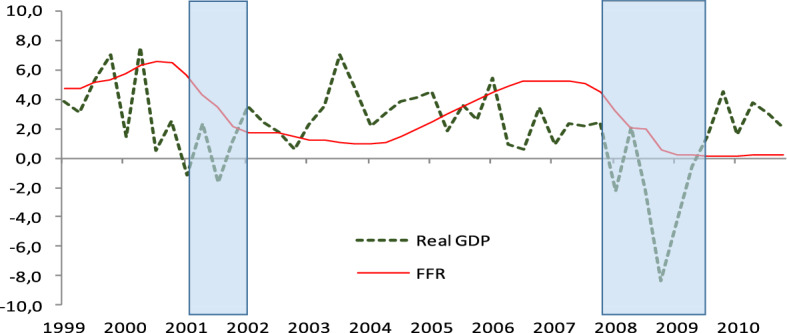
Real GDP (annual growth) and the Fed Funds Rate (FFR)

Figure 1 illustrates the annualized growth of real GDP between the past two recessions and the monetary policy responses reflected in the FFR. Clearly in this graph both real GDP changes and monetary policy are not uniform during the long expansion in between the two recessions, indicating that there is a shift from the early expansion phase of the business cycle to its late expansion period that usually takes place as a regime shift in monetary policy. However, the transition time from the early expansion to the late expansion period is not straightforward. We realize that refining the NBER U.S. business cycle methodology is a daunting task, thus we choose to offer empirical evidence that dividing an expansion into an early expansion and a late expansion that may overlap with an upper turning period, is supported by economic data. Our analysis focuses on this late expansion period where the economy has grown sufficiently to be close to full employment, with prices remaining stable, potential risks of inflation emerge and where the financial stability of the economy arises as a central issue. It is exactly this period that exhibits the complexity of the Fed’s decision making: should the Fed ignore its dual mandate and focuses on addressing risks to financial stability, or should it continue to pursue its traditional mandate at the expense of financial stability?

## Data and empirical models

### Data

We embed rich information from various dimensions of credit and financial markets. We choose monthly data series, from 1990:01 to 2018:08 instead of quarterly so we can have more detailed information about the evolution of the economy.

The financial variables we consider are grouped in three categories: credit, spreads and assets prices. Credit includes industrial loans (IL), household loans (HL) and credit to non-financial sector. Credit to non-financial sector is a very broad measure of credit provided by domestic banks, all other sectors of the economy and non-residents. The ‘non-financial sector’ includes non-financial corporations (both private-owned and public-owned), households and non-profit institutions serving households as defined in the System of National Accounts 2008. This is a quarterly series that is a much broader measure of credit than industrial or household loans.^2^ The spreads we consider are: the term spread that is the difference between the rate of the 10-Year Treasury Note and the 3-month Treasury Bill, it is a measure of longer term growth; the corporate bond spread defined as Moody’s seasoned Baa corporate bond yield rate less the yield on the 10-year Treasury Note constant maturity, this measures the default risk, and TED that is the difference between the 3-month LIBOR and the 3-month Treasury Bill that measures liquidity risk.

As we also wish to track the behavior of asset prices, we consider the CAPE (cyclically adjusted price to earnings ratio) as a measure of equity valuation and the S&P/Case-Shiller national home price index as a measure of home valuation. In addition, we augment our model by including the VIX index to capture financial volatility. Furthermore, exchange rate effects are captured by including the trade-weighted U.S. dollar index. We also account for the degree of consumer optimism regarding the consumers’ financial health and the health of the economy by considering consumer sentiment. Last, oil price is also included in the model given its ability to explain future economic growth and recessions (Kilian and Lewis, 2011). Regarding the macroeconomic variables, industrial production (IP) and consumer price index (CPI) are proxies for the central bank’s dual mandate of maximum growth and price stability while the FFR is the variable that denotes the monetary policy decided by the FOMC. All variables enter our empirical model in logs except the interest rates and spreads for which no transformation has been implemented. We call the model that includes all 15 variables as our full model.

### Large scale Bayesian VAR

VAR models treat all variables as a priori endogenous. Each endogenous variable is treated as a function of p-lagged values of itself and the other endogenous variables in the system. This means that the variables influence each other in a bi-directional relationship. Traditional VARs estimated either by least squares or maximum likelihood often require many lags to improve the in-sample fit, leading to a significant loss of degrees of freedom and thus to non-credible impulse responses and poor forecasts. Bayesian shrinkage, which is obtained by a prior that concentrates more around zero for higher lags allows us to reduce the number of lags hence, limiting the over-parameterization issue.

Furthermore, as Gambacorta et al. (2014) and Banbura et al. (2015) point out, a large set of variables capturing spillovers between the core macreconomy and the credit sector in needed to provide credible impulse responses and accurate forecasts of core macroeconomic variables. This is another limitation of the standard VAR models estimated by least squares or maximum likelihood and it is known as the ''curse of dimensionality”, that is, coefficients tend to increase exponentially with the number of endogenous variables and the number of lags. As several authors point out (Banbura et al. 2010, 2015; Koop and Korobilis 2010; Giannone et al. 2018), a common approach to deal with this complexity that arises due to large datasets is to use Bayesian VARs.^3^ This is because as explained above, Bayesian VARs implement parameter ‘shrinkage’, the intuition of which as nicely put by Banbura et al. (2015) is to bring together the likelihood from the highly parameterized VAR with a prior distribution for the parameters that is naive but enhances parsimony, thus, providing estimates that are ‘shrunk’ toward the prior expectations. Last, another advantage of using Bayesian VARs is that Bayesian simulation methods, such as Gibbs sampling that we use in this paper, provide an efficient way to obtain point estimates and to characterize the uncertainty around those point estimates by obtaining confidence bands. The structural VAR model is defined as:

1 $${y}_{t}=c+\sum_{j=1}^{p}{y}_{t-j}{B}_{j}+{\nu }_{t}$$

where ${y}_{t}$ is the matrix of endogenous variables, ${B}_{j}$ is the coefficient matrix, *c* is the vector of constant terms, and ${\nu }_{\mathrm{t}}\sim \mathrm{N}(0,\Sigma )$. The covariance matrix of the residuals, *Σ* can be decomposed as ${A}_{0}{A}_{0}^{^{\prime}}=\Sigma$, with ${A}_{0}$ representing the contemporaneous impact of the structural shocks,${\varepsilon }_{t}$*,* where ${\nu }_{t}={A}_{0}{\varepsilon }_{t}$*.* Based on standard information criteria, the lag length is set to four in the specification above.

In terms of identification, we follow a common approach that is adopted in the literature to disentangle credit supply from macroeconomic and monetary shocks through recursive identification and the Cholesky decomposition (Lown and Morgan 2006; Gilchrist and Zakrajsek 2012; Cesa-Bianchi et al. 2018). This means that the block of credit and financial variables, which are named as fast-moving variables, is placed second after the macro-variable block, which is the block of slow-moving variables. This ordering restricts shocks originating in financial markets from impacting the macroeconomic variables contemporaneously, while macroeconomic shocks are allowed to impact all variables within the same period. In particular, oil price is ordered first as the most exogenous variable, followed by IP, CPI and the credit to non-financial sector which is the proxy of credit supply; this is then followed by the group of loan and spread variables, asset prices indicators, volatility and sentiment indices, the FFR and the trade-weighted U.S. dollar index. The identifying assumption implied by this recursive ordering is that shocks to the credit to non-financial sector affect economic activity and inflation with a lag, while the rest of credit and financial variables in the system, as well as monetary policy, can react contemporaneously to such credit supply shocks. The quantitative and qualitative nature of the impulse responses to such a credit shock is robust to alternative orderings and identification schemes, in particular the adoption of sign restrictions which ensures that credit supply shocks are “uncontaminated” by credit demand shocks (see the Sect. 4.1.1).

In this paper, we follow Banbura et al. (2010), Blake and Mumtaz (2012), Evgenidis and Papadamou (2021) and Evgenidis and Fasianos (2021) who use a dummy observation prior to achieve Bayesian shrinkage. Intuitively, one can think of dummies in terms of artificial data featuring pseudo-observations for each of the regression coefficients with properties specified by the prior beliefs on the VAR parameters, and blending this with the real data. This prior is implemented as follows:

2 $${Y}_{D}=\left(\begin{array}{c}diag({\delta }_{1}{\sigma }_{1},\dots ,{\delta }_{n}{\sigma }_{n})/\lambda \\ {0}_{n\left(p-1\right)x n}\\ \dots \dots \dots \dots \dots \dots \dots \dots ..\\ diag\left({\sigma }_{1},\dots ,{\sigma }_{n}\right)\\ \dots \dots \dots \dots \dots \dots \dots \dots ..\\ {0}_{1xn}\end{array}\right){ X}_{D}=\left(\begin{array}{cc}{J}_{p}\otimes diag({\sigma }_{1},\dots ,{\sigma }_{n})/\lambda & {0}_{np x 1}\\ \dots \dots \dots \dots \dots \dots \dots \dots \dots .& \dots \dots ..\\ {0}_{n x np}& {0}_{n x 1}\\ \dots \dots \dots \dots \dots \dots \dots \dots \dots & \dots \dots ..\\ {0}_{1 x np}& c\end{array}\right)$$

where ${J}_{p}=diag(\mathrm{1,2},\dots ,p)$, ${\delta }_{i}$ is the prior mean of the coefficients on the first lags in the Minnesota prior; ${\sigma }_{i}$ denotes the standard deviation of the OLS residual obtained from individual autoregressive models, while $\lambda$ controls the overall tightness of the prior distribution and it reflects the relative importance of the prior beliefs with respect to the information contained in the data. Broadly speaking, the first block of dummies imposes prior beliefs on the autoregressive coefficients corresponding to the endogenous variables of the model, the second block implements the prior on the error covariance matrix, while the third block reflects the intercept which is set to a very small number.

We follow Banbura et al. (2010, 2015) and Blake and Mumtaz (2012) when setting the priors as they provide the optimal values for similar small and large-scale VARs. Starting with ${\delta }_{i}$, we perform an Augmented Dickey–Fuller (ADF) unit root test to verify which variables are stationary and which are not. Accordingly, we set ${\delta }_{i}=1$ for all variables that are characterized by high persistence while for stationary variables we set ${\delta }_{i}=0$. All our series are non-stationary, apart from the corporate bond spread and the VIX. The hyperparameter $\lambda$ is set in relation to the size of the VAR model. Practically, the higher the number of variables is, the more the parameters should be shrunk to avoid overfitting (this point has also been evidenced by De Mol et al. 2008). Last, it is worth noting that our dummy prior is consistent with the prior belief that variables can be represented by unit root and potential cointegrating processes. We use a Gibbs sampling algorithm to approximate the posterior distributions of the model parameters. The details of the estimation algorithm are described in the Appendix.

### Time varying VAR

We propose a novel way to empirically subdivide the expansion phase of the business cycle into an early and a late expansion phase, by studying how trends and volatilities of the shocks of core macroeconomic and financial variables that describe the US economy, have changed over time. Our goal is to verify whether early expansion phases and later expansion periods are distinct phases of the business cycle. This also justifies why we are using a model with time-varying coefficients and stochastic volatility.

Because TV-VAR models can be easily over-parametrized, a limited number of variables can be employed in this setting. The following core macro and key financial variables are used, the behavior of which will allow us to ascertain whether a differentiation between early and late expansion periods can be supported: industrial production, CPI, consumer sentiment, credit to non-financial sector and the policy rate, over the period 1990:01 to 2018:08. All variables enter as quarterly growth rates except for the policy rate which is simply incorporated in levels. We provide a description of the TV-VAR model and its estimation algorithm in the appendix.

## Results and discussion

This paper describes an articulate account of how disturbances in financial variables and the timing of monetary policy over the business cycle, shape the U.S. economy. The financial variables we consider are grouped into three categories that include credit variables, spreads between variables and asset prices. The FFR characterizes the monetary policy pursued by the central bank to attain its dual mandate of growth with price stability, denoted by industrial production and the consumer price index correspondingly.

As a synopsis of our central results, we highlight our three main findings. The first result traces the economic consequences of a positive credit supply shock. Such a shock is financial, and it immediately increases credit to the non-financial sector with economic effects on IP, interest rates, asset prices, inflation, risk aversion and financial uncertainty. This establishes that a financial shock has a significant impact on both real and financial economic variables. An increase in credit supply leads to a persistent and significant decline in economic activity in the medium term. Additionally, our model shows that the shock increases interest rates, asset prices and inflation but decreases risk aversion and financial uncertainty.

The second result establishes the forecasting capability of financial variables. Once we determine that a credit supply shock impacts both real and other financial variables, next we hypothesize that rapid credit growth is related to a higher likelihood of a financial crisis. We test this hypothesis by conducting several forecasting experiments to evaluate the predictive performance of various financial variables driven by economic theory and past empirical evidence. We discover a valuable result. The severity of the 2007–2009 Great Recession is most successfully predicted in our experiments, not by any single financial variable but jointly by few important financial variables including credit aggregates, asset prices and interest rate spreads.

The third result is about the mandate of financial stability to be pursued by the U.S. central bank. Having established the significance of financial variables in determining real and financial macroeconomic variables, and in addition, the predictive power of a subset of these financial variables, how can such findings be beneficial to monetary policy? The innovation introduced to obtain our third result is to apply a time-varying Bayesian VAR model to subdivide the business cycle into an early expansion phase and an upper turning period, so the central bank does not delay in taking action once the economy enters its upper turning period. Below, we expand in three subsections the discussion of key findings of our research.

### Financial shocks and the macroeconomy

**Fig. 2:**
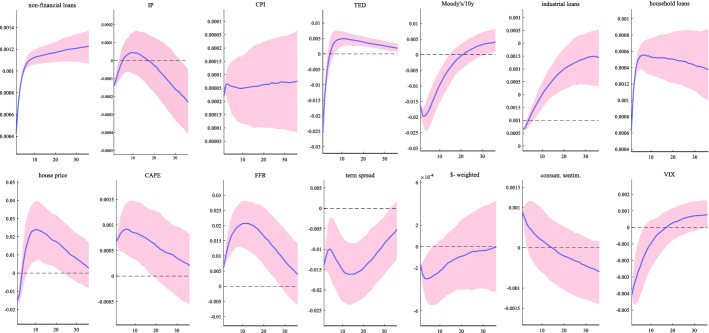
Impulse responses to a credit supply shock. Notes: The figure shows the impulse responses over 36 months of all variables to a positive credit supply shock. The blue line is the median estimate and the pink shaded area is the 68% error band

Figure 2 shows the impulse responses over 36 months of all variables to a positive credit supply shock. In our model, we assume a shock which increases credit to non-financial sector by 0.12% for the next 3 years. The shock has a small, short lived effect in depressing IP followed by an increase over the next 20 months that appears to be insignificant and finally, leading to a persistent and significant decline in economic activity. Our result appears to match the excessive credit growth narrative empirically supported by Brunnermeier et al. (2021) and Mian et al. (2017). The authors have demonstrated a predictive relation between rapid growth of credit and future low GDP growth by finding that an initial rise in GDP is followed by a subsequent decline that reaches about 0.1% after five years. In addition, our finding reconciles with the result from Aikman et al. (2020) who suggest that when credit growth is above trend, an impulse to financial conditions leads to an economic expansion in the near term, but then a recession in later quarters.

Regarding the impact on the other credit variables, our estimates show that interest rate spreads immediately decline in response to the shock, while the shock has a positive effect on credit aggregates and asset price indicators (as would be expected since a credit supply shock would typically lead to an opposite movement between credit spreads and lending). Relative to the impact of the credit supply shock to the rest of the variables in the system, we note a positive impact on inflation, a decrease of risk aversion and financial uncertainty (drop in VIX), while there is a short-term boost in consumer sentiment. The latter finding is consistent with Smales (2016) who finds that there is a significant positive relation between news sentiment and credit supply changes.

**Fig. 3:**
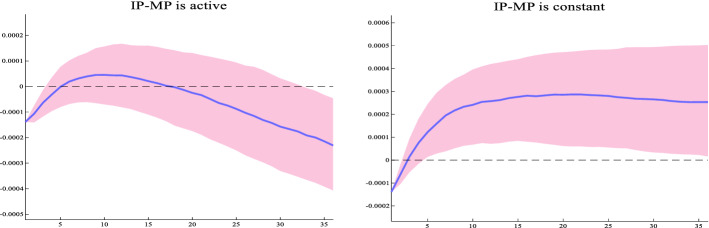
Impact of credit shock on IP when monetary policy is constant. Notes: The figure shows the impulse responses of IP to a positive credit supply shock. The left figure depicts the response of IP when monetary policy is active while the right figure depicts the response of IP assuming that the monetary policy response is zero. The blue line is the median estimate and the pink shaded area is the 68% error band

Note that the credit supply shock elicits a positive movement in FFR which is not surprising given that credit expansion is associated with rising inflation and a rising IP over the next 20 months. This means that the decline in economic activity observed in the medium term may be also due to the systematic monetary policy contraction that the credit supply shock evokes. To examine this, we run again our model by shutting down the monetary policy response. Specifically, we assume that the coefficients for the FFR in response to the credit shock are set to zero. Then, we compare the resulting constrained impulse response function for IP with the benchmark. Figure 3 shows that a constant interest rate policy eliminates the decline in IP in the medium term thus confirming the importance of monetary policy.

**Fig. 4:**
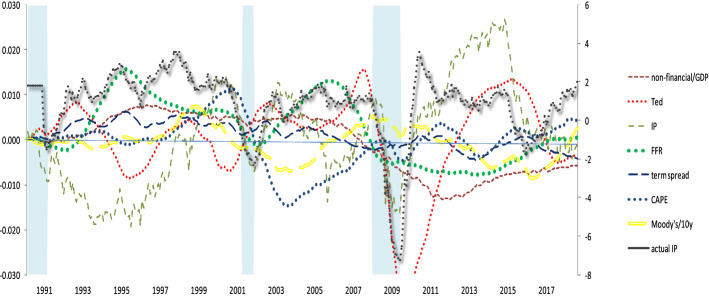
Historical decompositions-all shocks. Notes: The figure displays the historical decompositions of the fluctuations in IP. The black solid line shows the IP growth while the colourful lines depict the contribution of the various shocks along with NBER recessions (shaded areas)

Figure 4 displays the historical decompositions of the fluctuations in IP. Note that we show only the contributions of the most significant structural shocks to the changes in the forecast of IP. The figure show that besides industrial production shocks, the shocks of credit and asset price variables contribute substantially to the fluctuations of IP over the entire sample. Focusing on the factors that led to the Great Recession in 2007–2009, it appears that it is attributable to both interest rate spread shocks (banking and non-banking sector), monetary policy changes, asset price shocks and to a lesser extent credit supply shocks. The banking sector spread shock particularly contributes considerably not only to the period preceded the recession, but also during the 2007–2009 period.

In the years following the recession, credit supply shocks and the bank spread shock contribute significantly to the economic activity fluctuations while, as expected due to the zero lower bound period, it is worth mentioning the declining importance of monetary policy shocks. Finally, the importance of credit shocks in driving changes in the forecast of economic activity growth is also evidenced when we look at the first recession of 2000s. The interest rate spread and asset price shocks play a significant role in the 2001 recession which was triggered by the dot-com stock bubble. Last, the 2001 recession is mainly attributable to these two credit shocks and to a lesser extent to monetary policy shocks.

#### Robustness checks

We test the robustness of the main findings in Fig. 2 by implementing a series of robustness checks. We start by replicating the benchmark analysis by using three alternative identification schemes. The first two alternative identification strategies assume a different ordering of the variables. First, we replicate the benchmark analysis by ordering credit to non-financial sector last in the fast-moving variable block of the VAR. The implication is that credit and financial variables do not respond contemporaneously to credit supply shocks. Second, we order the asset price indicators after the credit to non-financial sector and before the spread variables. As the specific variables ordering within each block matters for contemporaneous relations, we also adopt sign restrictions as an additional robustness check. Sign restrictions on impulse responses have been frequently used in the literature to identify VAR structural shocks (Uhlig, 2005). We follow Busch et al. (2010), Gambetti and Musso (2017) and Furlanetto et al. (2017) by identifying a positive credit supply shock that raises credit to the non-financial sector and leads to a contemporaneous decrease in the credit spread and a rise in inflation, credit aggregates and asset prices. Note that we require that credit supply shocks move the credit spread, on the one hand, and the credit volumes, on the other hand, in opposite directions. This distinguishes credit supply shocks from credit demand shocks which would have moved corporate bond rates and the volume of credit in the same direction (Eickmeier and Ng, 2015; Furlanetto et al., 2017). In addition, the restrictions are imposed on the variables only on impact.

Second, we estimate two additional versions of the benchmark model, this time by checking whether our results are sensitive to the use of alternative macroeconomic measures of economic activity and inflation. Specifically, we re-estimate the benchmark model by replacing industrial production (IP) with the unemployment rate (first specification) and by replacing CPI with the producer price index (second specification). Third, we re-estimate our model by changing the number of lags included in the main specification. In particular, we consider alternative specifications with a lag length of two, three and five.

Overall, we find that the results (not depicted here but available upon request) under all these robustness checks are similar to those in the baseline shown in Fig. 2, with positive credit supply shocks generating a medium-term decline in IP, inflationary pressures and a positive response of the FFR rate.^4^

### Credit conditions and forecasting the recent financial crisis

Having demonstrated the importance of financial shocks in explaining economic activity fluctuations we next ask, are these credit and financial variables practically helpful to include in our model, and could this have been realized before the 2007–2008 financial crisis? In other words, is there any predictive relationship between a rapid growth of credit and a future higher likelihood of a recession?

There is a large literature that has attempted to examine the predictive power of credit growth, either using single-equation methods (e.g. Jorda et al. 2014, 2015) or binary predictive models (e.g. Drehmann and Juselius 2014; Ponka 2017; Mihai 2020), or small scale multivariate models with maximum three to four variables (e.g. Mian et al. 2017). Furthermore, Lopez-Salido et al. (2015) and Krishnamurthy and Muir (2016) using single-equation models, and, Gertler and Karadi (2015), Caldara and Herbst (2019) and Brunnermeier et al. (2021) using multiple equation models, incorporate credit spreads to investigate their forecasting power for predicting future low GDP. Del Negro and Schorfheide (2013) have used stochastic general equilibrium models augmented by financial frictions and with interest rate spreads to perform forecasts of the Great Recession. However, in contrast to our paper, all previous studies do not incorporate in their system the role of asset prices or/and credit aggregates and they have not considered as many financial and credit variables jointly in a medium scale multivariate time series model, as we consider here.

We conduct forecasting experiments to see what predictive value arises from including spreads, credit aggregate variables and asset prices in the system. To empirically investigate this, we consider the full model used in the previous section and four additional sub-models: (a) credit aggregates model which excludes spreads and asset prices, (b) spreads model which excludes credit aggregates and asset prices, (c) asset prices model that excludes credit aggregates and spreads and last, (d) a model without any credit variables.

We focus on the 2007–2008 financial crisis and its immediate aftermath. In particular, at each month between October 2007 and March 2008, we estimate posterior forecasts for all models using data only up to that point and then we calculate 12-month forecasts. These experiments allow us to compare models with different datasets and give us an understanding of the importance of credit variables in forecasting the recent financial crisis.

**Fig. 5:**
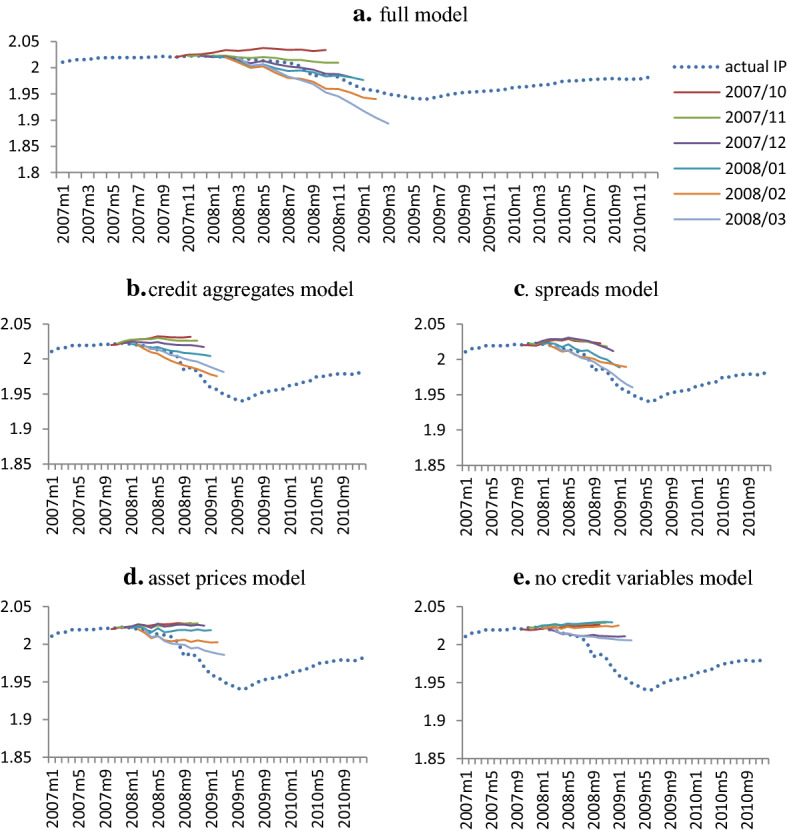
Posterior forecast distributions of IP. Notes: The figure shows the posterior forecast distributions (colourful lines) generated from various models. At each month between October 2007 and March 2008, we estimate posterior forecasts using data only up to that point and then we calculate 12-month forecasts. The blue dotted line shows the actual IP growth

Figure 5 shows the posterior forecast distributions (median) from the five different models. The full model is quick to recognize early recession signals. Specifically, the model provides worrying signals starting in November 2007 as evidenced by the curbing IP. The most important result however is the ability of the model to generate strong advance warning of the severe recession as evidenced by the forecasts from December 2007 up to March 2008. What is more, these forecasts are able to capture the magnitude and severity of the recession and its persistence well before the deepest contraction from mid-2008 to mid-2009.

To figure out whether the ability of the full model to provide early and strong recession signals is due to the impact of credit variables, we evaluate the forecasts of the other four models. First, all three models which contain subsets of credit variables that is, asset prices, credits spreads and credit aggregates, provide early optimistic forecasts (especially from January 2008 to March 2008) which warn us of the severe recession. Second, the fact that none of the three models alone is able to grasp the severity and persistence of the recession witnessed by the full model, highlights the necessity of jointly considering the information provided by various credit sources (interest rate spread, credit aggregates and asset prices) in order to grasp the severity of the crisis as captured by the full model. Third, when we consider the model without any credit variables, we observe that it does a poor job in forecasting the recession. The model’s forecasts never accept or recognize the crisis while the model consistently predicts that IP will return to near pre-crisis levels. Taken all these findings together, forecasts of IP are largely affected by excluding credit aggregates, interest rate spreads and asset prices. We show that the addition of credit variables can offer significant advance warning signals of the upcoming recession and enhance recognition of its severity.

Our results are in line with Caldara and Herbst (2019) who find that incorporating credit spreads explain about 20 percent of the volatility in industrial output and Brunnermeier et al. (2021) who show that the addition of credit spreads improves forecasts around the 2008 financial crisis in a narrow window at the beginning of the downturn and they do help in recognizing the severity of the recession once it has begun. The predictive power of credit aggregates in our system also reconciles with the results of previous researchers who have found significant predictive value for credit aggregates in forecasting future economic activity (Ponka 2017; Mian et al. 2017; Mihai 2020).

### Dual mandate, financial stability and lean against the wind

Four years after the Greenspan’s (1996) speech, the Fed experienced the crashing of the internet bubble that deflated NASDAQ by about 80% and caused a brief recession. Instead of reconsidering the policy “do not lean against a bubble”, a new policy was gradually formulated that proposed to “clean after the bubble bursts”. Mishkin (2011), Malliaris (2012), Evanoff et al. (2012) and recently Evgenidis and Malliaris (2020) give an extensive bibliographical overview of this debate and explain that the views held prior to the Global Financial Crisis of 2007–2008 favored the “clean” choice. Some key arguments for this choice and against the leaning approach included: First, strictly speaking the Fed’s dual mandate does not include management of asset bubbles. Second, it is very difficult to identify an asset price bubble from fundamentals; although it is true that some bubbles burst causing financial instabilities, others quietly deflate on their own. Third, the FFR is not an appropriate tool for deflating an asset bubble because an increase in the policy rate may cause a recession so the cost of deflating a bubble may be as high as the cost of the bursting of the bubble. However, the enormous financial and economic costs of cleaning after the Global Financial Crisis, contributed to a revision of the “clean” position in favor of the “lean” alternative.

Thus, the initial question that was raised in the mid-1990s about “leaning against the bubble or not” and had progressed to a consensus of “not leaning but instead cleaning after its bursting” as articulated by Blinder and Reis (2005), ended up in the painful realization that ignoring a housing bubble or a major asset bubble is not an unfailing answer.

#### The late expansion period

We propose a novel way to differentiate between early and late expansion periods and use this information (Sect. 4.3.2) in order to examine empirically possible initiatives by the central bank in terms of leaning against the growing bubbles. Specifically, we examine the dynamic properties of five key US macroeconomic and financial indicators over the last 30 years by using a TV-VAR with stochastic volatility, with a focus on the period in between the dot-com bubble and the 2007–2008 crisis. Our aim is to explore how trends and volatilities of shocks of these variables have changed over time in order to find out whether these changes can lead to a differentiation between early and late expansion periods.^5^

**Fig. 6:**
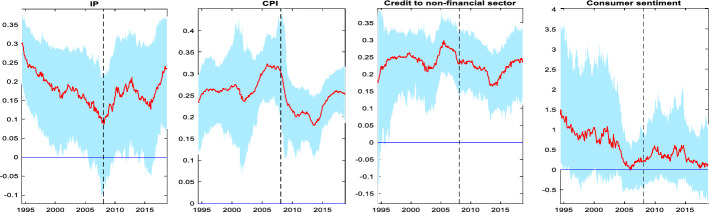
Time-varying long run means of the endogenous variables. Notes: The figure shows the time-varying unconditional means of the endogenous variables. The red lines are the median estimates while the light blue shaded areas represent the 68% error bands. The vertical dotted line denotes the beginning month of the last recession according to NBER

Figure 6 shows the unconditional means together with 68% confidence bands. The vertical dotted line denotes the beginning month of the last recession according to NBER. Overall, the estimates reveal that the series have experienced some notable changes in the period prior to the last recession with a clear distinction between the period before and after the first half of 2003. In particular, we see that the long-run mean of IP gradually rises in the period following the 2001 recession and stabilizes thereafter. From 2003 onwards however, the trend is reversed, and the long run mean of IP declines consistently and continuously, reaching a historical low in January 2008 (the recession began in December 2007 according to NBER). What is even more interesting to observe is that roughly at the same period (from 2003 onward), the long-run means of CPI and credit to non-financial sector show an exceptional rise that reaches the highest level during the first half of 2005 and stabilizes around this level for the next two years. On the other hand, we note a substantial drop in consumer sentiment that started in 2003 onward, revealing that the decline in the degree of confidence that consumers feel coincides with the period during which the log-run mean of IP falls notably and the log-run means of prices and credit growth exhibit a large and historical rise.

**Fig. 7:**
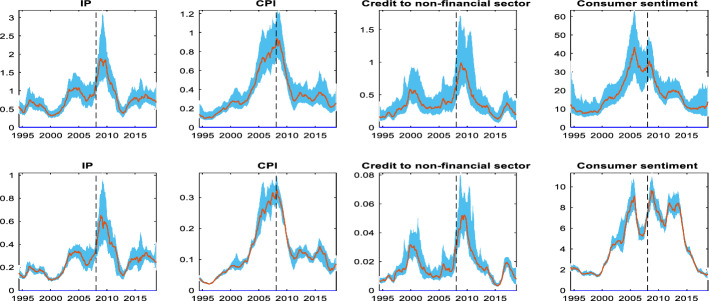
Time varying volatilities of the endogenous variables. Notes: The upper part of the figure shows the time-varying unconditional standard deviation of the endogenous variables. The lower part of the figure shows the time-varying stochastic volatility of the shocks. The red lines are the median estimates while the light blue shaded areas represent the 68% error bands. The vertical dotted line denotes the beginning month of the last recession according to NBER.

Figure 7 shows the dynamics of volatility as measured by our TV-VAR. The upper part of the figure shows the estimated unconditional standard deviation of each endogenous variable in the system while the bottom part shows the stochastic volatility of shocks. IP fluctuations do not seem to exhibit significant swings until the first half of 2003. At that point, we observe a notable change in this pattern as IP volatility shows an upward trend that reaches at a very high level in 2005. Since then, the IP volatility has remained at high levels despite a temporary fall which is finally followed by a sharp rise during the 2008–2009 period. These falls and rises in volatility coincide with comparable, but smaller in magnitude declines and increase in the volatility of IP shocks (lower part of the figure).

A similar pattern is observed for CPI. In particular, the volatility of CPI although upward trending, was fluctuating at relatively low levels in the sample period before 2003. From that year onwards, the upper part of the figure suggests that the volatility of CPI increases at a very fast rate, reaching to unprecedented high levels just before the onset of the last recession. This pattern is even clearer if one looks at the lower part of the figure, the volatility of CPI shocks. The upshot is that the increased volatilities of CPI and IP, coupled with the substantial drop in the long run mean of IP and the significant rise in the long run mean of prices after 2003, suggest that the Fed should have started raising the policy rate earlier than mid-2004.

Credit to non-financial sector increases significantly in the 2000–2001 recession period and stabilizes thereafter. For the period that we are interested in, that is, after the end of the 2001 recession and before the 2008 recession, we do not observe any notable changes in the volatility and the volatility of shocks of the credit to non-financial sector up to 2005; from that year onward however, we note that it gradually picks up. This low volatility between 2001 and 2005 indicates that markets had confidence that monetary policy will remain “easy” and it may be that in mid-2005, after GDP reached a very high rate, that markets started to get nervous. Last, although consumer sentiment volatility has increased steadily over time, one can notice the steady rise at very high levels from mid-2003 onward. The volatility of consumer sentiment remains elevated for this whole period until we notice the spike in 2008. Note that these volatility patterns are comparable to the volatilities of the two macroeconomic indicators, IP and CPI, for the same period. The upward trend in the volatility in the pre-2008 period coincides with the volatility of shocks to consumer sentiment during the same period.

Overall, in this section we witness clear evidence of two distinct sub-periods, before and after 2003. The long-run means of the core macroeconomic indicators reveal that there is a substantial drop in IP and a notable rise in inflation rate and credit, after 2003. Equally important, we note a significant fall of the consumer sentiment after 2003 indicating a pessimistic attitude towards future developments of the economy. Similarly, we find evidence of increases in the volatilities of IP, CPI and consumer sentiment after 2003. These dynamics of the volatilities (and the shock volatilities) can be roughly described as a transition from a low volatility regime (early expansion) to a high volatility regime (late expansion) between the period before and after 2003 correspondingly. Based on our results, we suggest that the early expansion could be supported by an easy monetary policy to help the Fed achieve its dual mandate, but as the economy recovers, markets realize that an easy monetary policy need not continue. It is this nervousness of markets anticipating a regime change by the Fed while at the same time the Fed is hesitant to undermine the early recovery fearing lower inflation and higher unemployment.

#### Is the central bank risk averse or a risk taker?

During the period prior to the recession of 2008, the Fed had delayed raising the policy rate, probably because they were worried that this policy would trigger spikes in inflation above the target of 2% and destabilized financial markets. As Fig. 1 shows, monetary policy began increasing the FFR in June 2004 in a monthly frequency and continued tightening until mid-2006. This policy might have been one of the factors that caused the financial instability the Fed tried to avoid. The lesson learned from this last time (but also the 2001 recession where the policy was similar) makes the current Fed very cautious in increasing rates continuously. Considering this, a natural question to ask is what would have happened if the Fed could go back at the dawn of the late expansion period, as identified in the previous section, and raised the FFR more gradually?

To estimate whether a policy of leaning against the build-up of financial imbalances could have lessened the consequences of the 2008 recession, we use our full model that incorporates linkages between credit variables and macroeconomic activity to carry out a counterfactual experiment that starts in January 2003 and ends in December 2009. Note that the design of the counterfactual experiment relies on the dichotomy between the expansion phase and the late period as identified in the previous section. In particular, we assume that at the dawn of the late expansion period, in January 2003, monetary policy starts to respond systematically to the imminent credit boom by increasing the FFR by 25 basis points per month, up to 5.25% in the mid of 2004 (June). After that date, the policy stabilizes at 5.25% until August 2007, where the FFR starts to fall. This last cycle of easing monetary policy (i.e. from August 2007 to December 2009) is consistent with the actual Fed policy which began lowering rates during that period, therefore, we assume that the counterfactual path during this particular period, follows the actual FFR path.

**Fig. 8:**
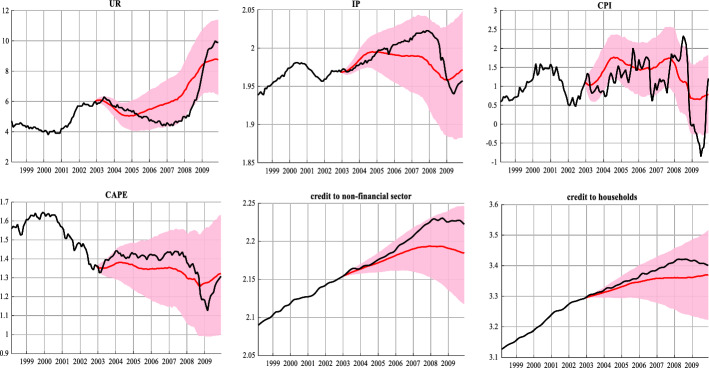
Counterfactuals: lean against the wind scenario. Notes: The figure depicts the counterfactual median estimates (red lines) for selected variables together with the 68% error bands (pink shaded area). The black line shows the actual data

The full model is used to simulate the economy one period ahead conditional on the counterfactual policy path. Note that we add unemployment as an extra variable in the vector of endogenous variables, as we are also interested in exploring potential costs by the leaning against the wind policy. Figure 8 shows the actual data (black line) and the results of our counterfactual experiment for IP, inflation, unemployment rate, CAPE, credit to households and credit to the non-financial sector (red lines). The results, consistent with Evgenidis and Malliaris (2020), suggest that leaning against the build-up of financial imbalances helps to moderate the increase in asset prices as well as credit growth in the period before the crisis (note the reduction in the counterfactual values of CAPE, credit to households and credit to non-financial sector, compared to their actual data). Additionally, although the counterfactual path of unemployment falls from 2003 to 2005, it starts rising thereafter to levels above the actual rate. This increase in unemployment observed after 2006 is not surprising given that the policy of leaning against the wind comes with the cost of higher unemployment.

The post-crisis (post-2008) counterfactual paths of IP, inflation and the unemployment rate offer additional evidence in favor of the lean against the wind hypothesis. Particularly, an early policy tightening against rising asset prices could have mitigated the severity of the 2008 recession as indicated by the counterfactual path of IP. Furthermore, note that during the recession, the US economy had fallen back into deflation (black line). However, our scenario shows that the counterfactual path of inflation never falls below zero which means that under this policy, the economy would not have experienced deflationary pressures. The gains become also apparent if one looks at the unemployment rate from 2009 to 2010. The counterfactual experiment points to a 2% lower unemployment rate than in the actual data. Taken together, these findings show evidence that, compared with the actual history, the alternative policy rule would yield noticeable output gains in the post crisis period, without significant costs in terms of scarring on employment, low inflation and growth effects of asset prices. Our results contradict earlier evidence by Svensson (2017) who concludes that the marginal costs of lean against the wind far exceed the benefits. Svensson’s conclusions however have been criticised by numerous studies for not properly accounting for systemic risk and the persistence of the financial cycle, thus ignoring the long-lasting effects financial crises may have on the economy. Our suggestion of leaning against the built-up of financial imbalances reconciles with more recent evidence by Kockerols and Kok (2019) who find that the marginal benefits of macroprudential policy outweigh the marginal costs.

## Conclusion

Dynamic interactions between credit conditions, the macroeconomic sector and monetary policy take place via multiple causal mechanisms. In this paper, we investigate these interactions by constructing and estimating two Bayesian VAR models that allow us to answer the following questions raised from the broadest to its narrowest scope.

First, what is the response of standard macroeconomic aggregates, credit and financial variables to credit supply shocks? We note that a shock that increases credit to non-financial sector has a short-lived effect in depressing IP, followed by an increase over the next 20 months, and leading to a persistent decline in economic activity. Regarding the impact on the other credit and financial variables, our estimates show that interest rate spreads immediately decline in response to the credit supply shock, while the shock has a positive effect on credit aggregates and asset price indicators. Furthermore, we find that the idiosyncratic shocks of credit and asset price variables contribute substantially to the fluctuations of industrial production over the entire sample, including both the 2001 and 2008 recessions.

Second, we ask whether the behavior of financial variables in the system is useful in signaling a recession? Our large scale BVAR that contains rich information from various dimensions of credit and financial markets can provide warning signals starting in November 2007, as evidenced by the curbing industrial production. Also, it is worth mentioning that the model generates strong advance warnings of the severe recession as shown by the IP forecasts from December 2007 up to March 2008. What is more noteworthy, these forecasts can capture the magnitude, severity of the recession as well as its persistence, well before the deepest contraction from mid-2008 to mid-2009.

Finally, we address the following policy relevant question: shall the central bank pursue its dual mandate at the expense of financial stability? In this paper we propose that policy makers need to consider business cycle phases when they design monetary policy. Specifically, our empirical evidence suggests that the expansion phase of the business cycle can be subdivided into an early and a late expansion, before and after 2003. Counterfactual experiments based on this separation indicate that if the Fed had resisted the fear of lower inflation and raised the policy rate earlier, that is in early 2003, when the economy moved from early recovery to late recovery, it could have significantly mitigated the severity of the 2008 recession without meaningful costs in terms of high unemployment, low inflation and growth effects of asset prices.

Taken together, our results suggest that credit plays an important role in determining the business cycle and the macroeconomy, including intensity of recessions, as suggested by the evidence we provide from the Great Recession. Our findings are very promising for two essential reasons. First, they confirm and extend evidence supplied by earlier studies on the importance of financial shocks in explaining the macroeconomy and second, they pinpoint the beneficial role of timing monetary policy to the expansion phases of the business cycle. Postponing the tightening of monetary policy to the very end of the expansion, makes such a policy more aggressive and thus less effective in terms of a steady financial stability.

Our findings are consistent with the aftermath of the Great Recession, where countries with faster credit growth, such as the UK and the U.S. in the run-up to the 2007/08 period, saw more sluggish recoveries than the economies of countries that went in with milder credit booms, such as Germany and the Emerging Markets. Similarly, the primacy of financial stability was amply demonstrated during the Covid-19 pandemic when the Fed on March 15 declared it was embarking on several emergency actions, most notably slashing its key interest rate to zero percent and launching an ambitious round of quantitative easing (QE) to ensure ample liquidity to the financial system.

Our results also suggest an appraisal of price stability, economic stability and financial stability as inter-related concepts. As the central bank addresses its mandates of price stability and full employment, these actions lead to economic stability. The financial sector provides the necessary liquidity for demand to grow so the economy operates on its potential output. Thus, financial stability is fully consistent with both price and economic stability. However, as the Global Financial Crisis illustrates, it is possible for the financial sector and asset prices to accelerate their increases as happened during the housing bubble, perhaps because monetary policy with its narrow focus on general inflation, did not get tighter earlier. The numerous papers we cited and our empirical evidence suggests that these three stabilities can easily diverge. Conceptually, we contribute to alerting central bankers to taking action earlier rather than later, by focusing on issues of financial stability.

Having pronounced the elevation of the financial sector as a means for enriching our understanding of macroeconomic outcomes and financial crises and having demonstrated empirically in our econometric experiments the central bank’s role in financial stability, we acknowledge that the consideration of an open economy and the impact of its global financial sector call for its analysis. Introducing a fourth stability, namely currency stability, will make the challenge more difficult.

In addition, alternative methods could be considered, for example Markov switching Bayesian VARs with time varying transition probabilities, to subdivide the expansion phase of the business cycle into an early expansion and a late expansion period and in this context, to investigate potential initiatives by the central bank to lean against a growing asset bubble. We leave this for future research.

Last, in terms of the scenarios that we construct in the last part of our paper to investigate whether a policy of leaning against the build-up of financial imbalances could have lessened the consequences of the 2008 recession; although they provide a new perspective from which to approach the policy problems, they are counterfactuals that are based on simple conditional forecasts. As this approach is necessarily more speculative, it faces the inevitable econometric limitations of the counterfactual experiment, a key one is that there is no presumption that the estimated coefficients are invariant to policy (Lucas’ critique). In order to interpret the BVAR scenarios in terms of structural shocks and derive policy implications, it may be useful to combine our Bayesian VAR model with a micro-founded, DSGE model, by feeding into the DSGE the projections for unemployment rate, inflation, output, the stock market (and possibly other observable variables), and derive implications for other variables that are of great interest for the lean against the wind analysis, such as the natural rate interest rate. This could be another interesting avenue for future research.

## Appendix

### Estimation of the Bayesian VAR

Define $${Y}^{*}=\left(\begin{array}{c}y\\ {Y}_{d}\end{array}\right)$$, $${X}^{*}=\left(\begin{array}{c}X\\ {X}_{d}\end{array}\right)$$ and ${T}^{*}=T+{T}_{d}$. That is, *Y*^***^and *X*^***^ are obtained by concatenating the dummy observation matrices at the top of the actual data matrices *Y* and *X*, and *T* ^*^ is the total number of time periods, obtained from adding the actual and simulated time periods. The Gibbs sampling algorithm consists of the following steps:

*Step 1*: Sample the VAR coefficients from their posterior normal distribution

$$H\left(B\backslash\Sigma ,{Y}_{t}^{*}\right)\sim N\left({\mu }^{*}, {n}^{*}\right)$$

where:

$${\mu }^{*}={\left({\Sigma }^{-1}\otimes {\left({X}_{t}^{*}\right)}^{^{\prime}}{X}_{t}^{*}\right)}^{-1}\left({\Sigma }^{-1}\otimes {\left({X}_{t}^{*}\right)}^{^{\prime}}{Y}_{t}^{*}\right)$$

$${n}^{*}={\left({\Sigma }^{-1}\otimes {\left({X}_{t}^{*}\right)}^{^{\prime}}{X}_{t}^{*}\right)}^{-1}$$

*Step 2:* Given B from step 1, draw $\Sigma$ from its posterior, inverse Wishart, distribution

$$H\left(\Sigma \backslash B,{Y}_{t}^{*}\right)\sim IW\left(\overline{\Sigma },{\rm T}^{*}\right)$$

where:

$$\overline{\Sigma }={\left({Y}_{t}^{*}-{X}_{t}^{*}B\right)}^{^{\prime}}\left({Y}_{t}^{*}-{X}_{t}^{*}B\right)$$

### Time-varying VAR model

Consider again the structural VAR in Eq. (1):

$${y}_{t}=c+\sum_{j=1}^{p}{y}_{t-j}{B}_{j}+{\nu }_{t}$$

but now following Primicery (2005), the covariance matrix *Σ*_*t*_ is decomposed as:

$${\Omega }_{t}={A}_{t}^{-1}{H}_{t}{\left({A}_{t}^{-1}\right)}^{^{\prime}}$$

In our five VAR specification, the time-varying matrices ${H}_{t}$ and ${A}_{t}$ are defined as follows:

$$H_{t} = \left[ {\begin{array}{*{20}c} {h_{1,t} } & 0 & 0 & 0 & 0 \\ 0 & {h_{2,t} } & 0 & 0 & 0 \\ 0 & 0 & {h_{3,t} } & 0 & 0 \\ 0 & 0 & 0 & {h_{4,t} } & 0 \\ 0 & 0 & 0 & 0 & {h_{5,t} } \\ \end{array} } \right]\,{\text{and}}\,A_{t} = \left[ {\begin{array}{*{20}c} 1 & 0 & 0 & 0 & 0 \\ {a_{21,t} } & 1 & 0 & 0 & 0 \\ {a_{31,t} } & {a_{32,t} } & 1 & 0 & 0 \\ {a_{41,t} } & {a_{42,t} } & {a_{43,t} } & 1 & 0 \\ {a_{51,t} } & {a_{52,t} } & {a_{53,t} } & {a_{54,t} } & 1 \\ \end{array} } \right]$$

where ${H}_{t}$ is a diagonal matrix of the stochastic volatilities and ${A}_{t}$ is a lower triangular matrix which captures the contemporaneous interactions of the endogenous variables. Following Primiceri (2005), the elements of ${B}_{i,t}, {h}_{i,t},{a}_{ii,t}$ are modeled as random walks. The advantage of this approach is that we allow for permanent shifts while we reduce the number of parameters to be estimated in a model which is already heavily parameterized. In particular, denoting ${h}_{t}={\left[{h}_{1,t},{h}_{2,t},{h}_{3,t},{h}_{4,t},{h}_{5,t}\right]}^{^{\prime}}$ and ${a}_{t}={\left[{a}_{21,t},{a}_{31,t},\dots {a}_{54,t}\right]}^{^{\prime}}$, we have that:

$${lnh}_{t}={lnh}_{t-1}+{n}_{t}$$

$${B}_{t}={B}_{t-1}+{\eta }_{t}$$

$${a}_{t}={a}_{t-1}+{\tau }_{t}$$

where ${h}_{i,t}$ evolves as a geometric random walk and ${B}_{t}$, ${a}_{t}$ evolve as driftless random walks. We assume that the vector ${[{\varepsilon }_{t},{\eta }_{t},{\tau }_{t},{\nu }_{t}] }^{^{\prime}}$ is distributed as:

$$\left[\begin{array}{c}{v}_{t}\\ {\eta }_{t}\\ {\tau }_{t}\\ {n}_{t}\end{array}\right]\sim N\left(0,V\right), \mathrm{with} V=\left[\begin{array}{cccc}\Sigma & 0& 0& 0\\ 0& Q& 0& 0\\ 0& 0& S& 0\\ 0& 0& 0& Z\end{array}\right]$$

As before, the model is estimated by using Bayesian methods. Specifically, we employ a Gibbs algorithm that approximates the posterior distributions of the parameters (see Sect. A.2.2).

As we note in the main text, the time-varying VAR model is used to examine the trends and volatilities of the variables to detect potential changes over time. In particular, considering the TV-VAR model above, the unconditional means of each variable can be estimated as the local linear approximation:

$$E\left({y}_{t}\right)={e}_{N}{(I-{B}_{t})}^{-1}c$$

where ${e}_{N}$ is a selection matrix that picks out the first *N* elements of $E\left({y}_{t}\right)$ where *N* the number of variables included in the TV-VAR. Next, the unconditional standard deviation of each variable is approximated as:

$$\sigma \left({y}_{t}\right)=\sqrt{{e}_{N}{\left(I-{B}_{t}\otimes {B}_{t}\right)}^{-1}vec\left(\Sigma \right)}$$

#### Prior distributions

The initial conditions for the VAR coefficients *B*_*0*_ are obtained via an OLS estimate of a fixed VAR using the first T_0_ observations and then the prior distribution for *B*_*0*_ is defined as ${B}_{0}\sim N\left[{\widehat{B}}_{OLS,},4x\widehat{V}\left({\widehat{B}}_{OLS}\right)\right]$. For the prior of *h*, let ${\widehat{\Sigma }}_{OLS}$ be the estimated covariance matrix of ${\nu }_{t}$ from the estimation of the time-invariant version of (1) and let *K* be the lower triangular Choleski factor under which $K{K}^{^{\prime}}$=${\widehat{\Sigma }}_{OLS}$*.* The prior is then defined as ${lnh}_{0}\sim N\left({ln\mu }_{0},10x{I}_{3}\right)$ where ${\mu }_{0}$ is a vector collecting the logarithms of the squared elements on the diagonal of *K*. For the prior of the off-diagonal elements of *A*, we divide each column of *K* by the corresponding element on the diagonal and then we define ${a}_{0}\sim N\left[{\tilde{a }}_{0},\tilde{V }({\tilde{a }}_{0})\right]$ where ${\tilde{a }}_{0}={\left[{\tilde{a }}_{\mathrm{0,1}},{\tilde{a }}_{\mathrm{0,2}},{\dots \tilde{a }}_{\mathrm{0,6}}\right]}^{^{\prime}}$ is a vector containing all the zero and non-one elements of ${K}^{-1}$ and $\tilde{V }({\tilde{a }}_{0})$ is a diagonal matrix with each element *(i,i)* being 10 times the absolute value of the corresponding *i-th* element.

Regarding the prior distributions for the hyperparameters, the prior of $$Q$$ is assumed to be inverse Wishart distribution $$Q\sim IW\left( {T_{0} \overset{\lower0.5em\hbox{$\smash{\scriptscriptstyle\smile}$}}{Q} ,T_{0} } \right).$$ The scale parameter is equal to $$T_{0} \overset{\lower0.5em\hbox{$\smash{\scriptscriptstyle\smile}$}}{Q}$$, where $$\overset{\lower0.5em\hbox{$\smash{\scriptscriptstyle\smile}$}}{Q} = \rho x\hat{\Sigma }_{{OLS}}$$*,* and $$\rho =0.0001$$. The prior distribution of the elements of *S* is assumed to be inverse Wishart $${S}_{i}\sim IW\left({S}_{{\mu 0}_{i}},{S}_{{v0}_{i}}\right)$$ where $$i$$ indexes the blocks of *S* where $${S}_{{\mu 0}_{i}}$$ is a diagonal matrix with the relevant elements of $${\tilde{a }}_{0}$$ multiplied by 10^–3^. Finally, for the variances of the stochastic volatility innovations, we set an inverse Gamma distribution for the elements of *Z,* $${\sigma }_{i}^{2}\sim IG\left({\sigma }_{\mu 0}=\frac{0.0001}{2},{\sigma }_{\sigma 0}=\frac{1}{2}\right)$$

#### Estimation algorithm for the TV-VAR model

A Gibbs sampling algorithm is used to sample from the posterior distribution. The details of each conditional distribution are provided below.

##### 1st step; drawing the coefficient states ${B}_{t}$

Conditional on ${A}_{t}, {H}_{t} V$, the observation Eq. (1) is linear with Gaussian innovations and a known covariance matrix. Therefore, we draw ${B}_{t}$ using the Carter and Kohn (1994) algorithm as follows. The conditional posterior distribution of $p\left({B}^{T}\setminus {Y}^{T},{A}^{T}, {H}^{T}, V\right)$ is written as $p\left({B}^{T}\setminus {Y}^{T},{A}^{T}, {H}^{T}, V\right)=p\left({B}_{T}\setminus {Y}^{T},{A}^{T}, {H}^{T}, V\right)\prod_{t=1}^{T-1}p({B}_{t}\setminus {B}_{t+1},{Y}^{T},{A}^{T}, {H}^{T}, V)$. The first term on the right hand side equation, i.e. the posterior distribution of *B*_*T*_ is distributed as.

$p\left({B}_{T}\setminus {Y}^{T},{A}^{T}, {H}^{T}, V\right)\sim N\left({B}_{T|{\rm T}},{P}_{T|{\rm T}}\right)$*.* The second element, i.e. the posterior distribution of ${B}_{T}$ is distributed as $p\left({B}_{t}\setminus {B}_{t+1},{Y}^{T},{A}^{T}, {H}^{T}, V\right)\sim N\left({B}_{t\setminus t+1},{P}_{t\setminus t+1}\right).$ The simulation proceeds as follows. First we use Kalman filter to draw ${B}_{T|{\rm T}},{P}_{T|{\rm T}}$ and then we proceed backwards in time by using ${B}_{t\setminus t+1}={B}_{t\setminus t}+{P}_{t\setminus t}{P}_{t\setminus t+1}^{-1}\left({B}_{t+1}-{B}_{t}\right)$ and ${P}_{t\setminus t+1}={P}_{t\setminus t}+{P}_{t\setminus t}{P}_{t\setminus t+1}^{-1}{P}_{t\setminus t}$.

##### 2nd step; draw the covariance states ${a}_{i}$

Before describing this step, note that${\nu }_{t}$, the VAR residuals, can be written as ${{A}_{t}\nu }_{t}={\varepsilon }_{t}$ with$var({\varepsilon }_{t})={H}_{t}$. This is a system of linear equations with time varying coefficients and heteroskedasticity which has a known form. The *j*_*th*_ equation of this system is given as ${\nu }_{jt}=-{a}_{jt}{\nu }_{-jt}+{\varepsilon }_{jt}$, where the subscript *j* denotes the *j*_*th*_ column of${\nu }_{t}$, while *-j* denotes columns 1 to *j-1*. Note that this is a system of equations with time varying coefficients *α*_*t*_. Following Primiceri (2005), we simplify the analysis by allowing the covariance of${\tau }_{t}$, *S*, to be block diagonal, that is, the shocks to the *j*_*th*_ equation ${\tau }_{j,t}$ are uncorrelated with those from other equations. Given this diagonal form, the elements of ${A}_{t}$ can be drawn by using the standard Carter and Kohn (1994) algorithm.

##### 3rd step; draw the volatility states, *h*_*t*_

Following Cogley and Sargent (2005), the diagonal elements of ${H}_{t}$ are sampled using a Metropolis Hastings algorithm. To see this, we write the following equation as before, ${{A}_{t}\nu }_{t}={\varepsilon }_{t}$, where, $var({\varepsilon }_{t})={H}_{t}$. Now, conditional on ${B}_{t}$ and ${A}_{t}$, the distribution *h*_*it*_ is given by:

$$\begin{aligned} f(h_{it} /h_{it - 1} , h_{i,t + 1, } \varepsilon_{it} ) & = f\left( {\varepsilon_{it} \backslash h_{it} } \right) x f(h_{it} \backslash h_{it - 1} ) x f\left( {h_{it + 1} \backslash h_{it} } \right) \\ & = h_{it}^{ - 0.5} exp\left( {\frac{{ - \varepsilon_{it}^{2} }}{{2h_{it} }}} \right) x h_{it}^{ - 1} \exp \left( {\frac{{ - \left( {lnh_{it} - \mu } \right)^{2} }}{{2\sigma_{{h_{i} }} }}} \right) \\ \end{aligned}$$

where *μ* and ${\sigma }_{{h}_{i}}$ denote the mean and variance of the log-normal density ${h}_{it}^{-1}\mathrm{exp}\left(\frac{-{(ln{h}_{it}-\mu )}^{2}}{2{\sigma }_{{h}_{i}}}\right)$. Following Jacquier et al. (1994), we use this log normal density as the candidate generating density with the acceptance probability defined as the ratio of the conditional likelihood ${h}_{it}^{-0.5}exp\left(\frac{-{\varepsilon }_{it}^{2}}{2{h}_{it}}\right)$ at the old and the new draw. This algorithm is applied at each period in the sample to deliver a draw of the stochastic volatilities.

##### 4th step, draw the hyperparameters, *Q*, *S*, *Z*

Conditional on *B*_*t*_*, A*_*t*_*, H*_*t*_, we sample the hyperparameters as follows: *Q* is sampled from the inverse Wishart distribution using the scale matrix ${\eta }_{t}^{^{\prime}}{\eta }_{t}+{Q}_{0}$ and degrees of freedom *T* + *T*_*0*._ Next, *S* is sampled from the inverse Gamma distribution with scale parameter ${{\tau }_{t}^{^{\prime}}{\tau }_{t}+S}_{i}$ and degrees of freedom *T* + *T*_*0*_. Last, we draw the elements of *Z* from its inverse Wishart distribution with scale parameter $\frac{{\left(ln{h}_{it}-ln{h}_{it-1}\right)}^{^{\prime}}\left(ln{h}_{it}-ln{h}_{it-1}\right)+{\sigma }_{\mu 0}}{2}$ and degrees of freedom,$\frac{T+{\sigma }_{\sigma 0}}{2}$.

## Footnotes

1 See http://www.nber.org/cycles.html.

2 Credit to non-financial sector data is converted into monthly frequency through liner interpolation.

3 Related to the size of the VARs, as Christiano, Eichenbaum, and Evans (1999) and Giannone and Reichlin (2006) point out, the size of VARs that is used in macroeconomic applications plays an important role as, a model with a limited number of variables (≤ 5) could create an omitted variable bias with adverse consequences for impulse responses analysis and forecasting. In support of this, Banbura, Giannone and Reichlin (2010) show that by estimating BVARs of a similar size as the one used in our paper, they produce credible impulse responses and better forecasting results than standard small-scale VARs that typically considered in the literature.

4 In terms of our alternative specification that includes the unemployment rate, note that consistent with the response of IP, we find that a positive credit supply shock is associated with higher subsequent unemployment. This result is in line with Mian et al. (2017).

5 We use the Matlab code in Akram and Mumtaz (2019) to derive trends and volatilities.
